# Diffusion microscopic MRI of the mouse embryo: Protocol and practical implementation in the *splotch* mouse model

**DOI:** 10.1002/mrm.25145

**Published:** 2014-03-13

**Authors:** Francesca C. Norris, Bernard M. Siow, Jon O. Cleary, Jack A. Wells, Sandra C.P. De Castro, Roger J. Ordidge, Nicholas D.E. Greene, Andrew J. Copp, Peter J. Scambler, Daniel. C. Alexander, Mark F. Lythgoe

**Affiliations:** ^1^ UCL Centre for Advanced Biomedical Imaging, Division of MedicineUniversity College LondonLondonUnited Kingdom; ^2^ Centre for Mathematics and Physics in the Life Sciences and EXperimental Biology (CoMPLEX)University College LondonLondonUnited Kingdom; ^3^ Centre for Medical Image Computing, Departments of Medical Physics and Bioengineering and Computer ScienceUniversity College LondonUnited Kingdom; ^4^ Department of Anatomy and NeuroscienceUniversity of MelbourneAustralia; ^5^ Neural Development Unit, UCL Institute of Child HealthUniversity College LondonLondonUnited Kingdom; ^6^ Molecular Medicine Unit, UCL Institute of Child HealthUniversity College LondonLondonUnited Kingdom

**Keywords:** phenotyping, diffusion microscopic magnetic resonance imaging, mouse embryo, splotch mouse model, spina bifida

## Abstract

**Purpose:**

Advanced methodologies for visualizing novel tissue contrast are essential for phenotyping the ever‐increasing number of mutant mouse embryos being generated. Although diffusion microscopic MRI (μMRI) has been used to phenotype embryos, widespread routine use is limited by extended scanning times, and there is no established experimental procedure ensuring optimal data acquisition.

**Methods:**

We developed two protocols for designing experimental procedures for diffusion μMRI of mouse embryos, which take into account the effect of embryo preparation and pulse sequence parameters on resulting data. We applied our protocols to an investigation of the *splotch* mouse model as an example implementation.

**Results:**

The protocols provide DTI data in 24 min per direction at 75 μm isotropic using a three‐dimensional fast spin‐echo sequence, enabling preliminary imaging in 3 h (6 directions plus one unweighted measurement), or detailed imaging in 9 h (42 directions plus six unweighted measurements). Application to the *splotch* model enabled assessment of spinal cord pathology.

**Conclusion:**

We present guidelines for designing diffusion μMRI experiments, which may be adapted for different studies and research facilities. As they are suitable for routine use and may be readily implemented, we hope they will be adopted by the phenotyping community. **Magn Reson Med 73:731–739, 2015. © 2014 The Authors. Magnetic Resonance in Medicine published by Wiley Periodicals, Inc. on behalf of International Society for Magnetic Resonance in Medicine. This is an open access article under the terms of the Creative Commons Attribution License, which permits use, distribution and reproduction in any medium, provided the originalwork is properly cited.**

## INTRODUCTION

Worldwide collaborative efforts 1, 2 are underway to develop a comprehensive functional annotation of the mouse genome, which will serve as a rich resource for elucidating human gene function. At least one third of all protein‐encoding genes in the mouse genome are essential for development 3, and mutagenesis programs have identified many genetically engineered mutations that result in embryonic lethality 4. As complete inactivation of these genes will preclude further investigation in adult mice, a dedicated mouse embryo screening pipeline is urgently needed to characterize the anatomical phenotype of the increasing number of mutant embryos being generated 5. Furthermore, a dedicated screening pipeline will facilitate general mouse embryo phenotyping studies, such as investigations of the genetic causes of congenital abnormalities, and provide primary evidence to justify detailed follow‐up studies. This will give insight into the functional and structural consequences of gene inactivation in developmental processes, as well as better models of human diseases and novel therapies.

Imaging platforms that enable detailed phenotyping include optical projection tomography (OPT) 6, 7, microscopic computed tomography (μCT) 8, 9, microscopic MRI (μMRI) 10, 11 and high‐resolution episcopic microscopy (HREM) 12, 13. These modalities will most likely form the backbone of the screening pipeline as a complement to conventional optical histological examination, enabling acquisition of digital, high‐resolution, three‐dimensional (3D) data. To supplement this pool of techniques, advanced methodologies that enable visualization of novel tissue contrast will be essential for in‐depth screening and assessment of specific pathologies.

In particular, μMRI has been widely used in embryo phenotyping studies since its first application in 1986 14, offering high‐throughput and nondestructive screening capabilities 15, 16, 17, 3D isotropic datasets with high resolution [∼18 μm 18, 19], and morphometric methods of analyses 20, 21, 22. While conventional relaxation‐based MRI approaches may be used to identify structural malformations 16, 20, 23, they provide limited tissue contrast in the developing mouse brain, largely due to the lack of myelination 24, 25, 26. MRI contrast agents are commonly incorporated in the tissue fixation process to enhance contrast, boost the signal‐to‐noise ratio (SNR), and reduce scanning times by significantly lowering the tissue T_1_ relaxation time. For example, Gd‐DTPA (gadolinium diethylene triamine pentaacetic acid) has been used in mouse embryo phenotyping studies to visualize vasculature 10, 27 and gross anatomy 16, 19, 20, 23, 28, and has been shown to provide some discrimination of developing white‐matter structures 20. However, the specificity and sensitivity of the wide range of available agents have yet to be fully explored in the embryo 29.

Diffusion MRI is a powerful methodology 30 for noninvasively probing tissue microstructure. As the diffusion MRI signal is dependent on the dispersion pattern of water molecules, it is sensitive to the presence and orientation of ordered tissue structures. Thus, it can provide unique neuroanatomical information about the premyelinated embryo central nervous system (CNS), such as discrete delineation of multiple cortical layers, gray matter structures, and various white matter tracts 24, 25, 26. This has enabled volumetric analysis of whole brain and substructures 31, and facilitated 3D mapping of gene expression data 25. Accordingly, diffusion MRI, and in particular diffusion tensor imaging (DTI), has contributed to the characterization of normal brain development at multiple stages 21, 26, 31, 32 and enhanced phenotypic assessment of mutant embryos 33, 34, 35.

Although DTI has been used successfully to assess the phenotypic characteristics of mutant embryos in several studies, practical implementation of this technique in an embryonic screening pipeline and widespread routine use of DTI for embryo phenotyping have been restricted by an extended scanning time—an order of magnitude higher than conventional structural imaging—which is partly due to limited methodological investigation. DTI studies in the developing mouse brain have typically minimized the scanning time by using 16 or fewer unique sampling directions. However, a Monte Carlo simulation has shown that the ideal number of directions is at least 30 36, and under‐sampling could compromise the robustness of common DTI measures, including the principal eigenvector, mean diffusivity (MD) and fractional anisotropy (FA). This may also preclude the use of advanced diffusion MRI techniques, such as spherical deconvolution 37, 38, 39, super resolution 40, 41, and the apparent fiber density 42. Furthermore, there is currently no established protocol for mouse embryo phenotyping using μDTI that ensures optimal data acquisition for any user. An approach that can accommodate different needs and MRI hardware systems would be useful for providing more robust comparisons of data between research facilities, such as the MD and FA, which are dependent on the scanning parameters (e.g., diffusion time). For example, there is a large variation in the experimental set‐up to control sample temperature, whether passive, intrinsic, or extrinsic, as the gradient subsystem set‐up varies in the diameter of the unit and efficiency of the gradient cooling. Thus, these factors need to be considered during the experimental design stage.

We present two protocols for phenotypic characterization of embryos using diffusion μMRI, which provides users with an experimental procedure for their particular diffusion μMRI study requirements, MRI system hardware and experimental design. Building on previous work 23, 24, 25, 30, 31, 32, 33, 34, we have developed protocols that enable data acquisition in a reasonable timeframe with a standard imaging sequence, such that this technique may be readily implemented in an embryonic imaging pipeline and by individual researchers. As the protocols initially account for the choice of diffusion protocol and MRI hardware, which can affect the embryo preparation by means of sample heating and confound resulting data, it provides adaptability and flexibility for different phenotyping studies and research facilities.

As a demonstration, we also implement our protocols in an example application: a whole‐body phenotyping study that examines the wider effects of spina bifida on the developing CNS. In particular, we investigate the *splotch* mouse model of human neural tube defects (NTDs) 43. NTDs are a common group of congenital malformations, including spina bifida and exencephaly, which affect 0.5–2 per 1000 pregnancies worldwide 44. These mice carry a mutation in the *Pax3* (paired box 3) gene, a transcription factor implicated in these conditions 45, 46. For this example application, we applied a two‐stage experiment: a short diffusion protocol and a more extensive detailed diffusion protocol. The short diffusion protocol uses six directions at 75 μm isotopic, providing a ∼3 h data acquisition time per embryo, such that it may be used for preliminary investigations. More accurate DTI measures [35] were acquired for detailed imaging using an extensive detailed diffusion protocol, which used 42 directions at 75 μm isotopic, providing a ∼19 h data acquisition time per embryo.

## METHODS

### Diffusion μMRI Investigations: Short and Long Diffusion Protocols

**Figure 1:**
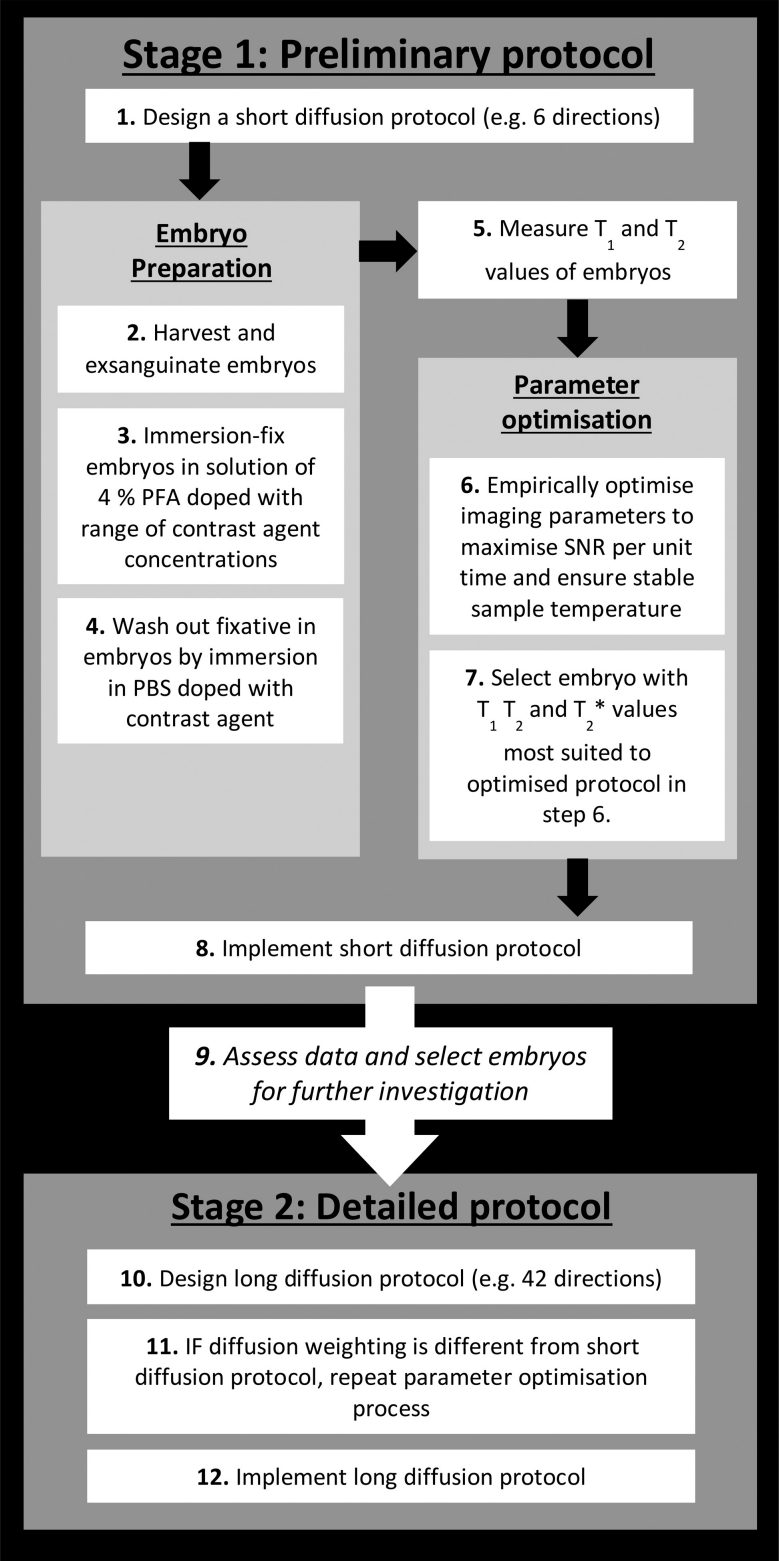
Schematic of the protocol used in creating our diffusion μMRI experimental procedure.

Based on our experience, we have developed methods to enable a two‐stage experimental procedure for implementing diffusion μMRI of mouse embryos (Fig. 1), which provides preliminary and/or detailed diffusion data. The first step is to select a short diffusion protocol (short diffusion weighted [DW] protocol) for relatively fast preliminary imaging (e.g., six directions at a defined b‐value). Subsequently, the MR imaging parameters (e.g., repetition time [TR]) and experimental setup are then empirically optimized to ensure that the protocol does not cause sample heating and maximizes SNR, while ensuring image quality. Contrast agent concentration and fixative washout duration is then selected to provide the optimal combination of T_1_ and T_2_ for the desired protocol, experimental setup and MRI hardware. Upon implementation of the short DW protocol and assessment of the data, a more extensive diffusion protocol (long DW protocol) may be adopted for more detailed investigation of embryos. The imaging parameter optimization process is repeated before implementation if a different diffusion weighting is used.

### Embryo Preparation

All animal work was performed according to protocols approved by the UK Home Office. Time‐mated wild‐type (C57BL/6) mice were killed at 15.5 days post coitum (dpc) by cervical dislocation, and the embryos were dissected from the uterus. The umbilical cords were cut close to the placenta allowing the embryos to exsanguinate in Hanks' solution at 37°C. A small incision (∼ 2 mm) was made in the side of each embryo, just below the liver, to facilitate penetration of the contrast agent. The embryos were immersion‐fixed in a solution of 4% PFA (4% aqueous formaldehyde from paraformaldehyde) and stored at 4°C.

### Data Acquisition

A 9.4 Tesla (T) magnetic resonance imaging system (Agilent Technologies Inc., Palo Alto, CA) was used with a 1T/m gradient set (60 mm diameter, with a water‐based gradient cooling unit, set to 17°C) and 26 mm volume coil (RAPID Biomedical GmbH, Würzburg, Germany). For scanning purposes, each embryo was placed in a sealed 10‐mL syringe filled with Perfluorosolv PFS‐1 solution (Solvay Solexis Inc., West Deptford, NJ), which prevents dehydration and provides susceptibility matching. Each embryo was carefully secured in place with the plunger, using gauze for additional padding. The sample temperature was monitored throughout all acquisitions using an MR‐compatible temperature probe designed for physiological monitoring (SA Instruments Inc., Stony Brook, NY).

### Tissue Temperature Equilibrium

Preliminary experiments were conducted with and without the MRI diffusion weighting gradients to determine the main source of sample heating. As diffusivity changes with temperature 47, the temperature of an unstained embryo was monitored at different TR values during diffusion‐weighted (DW) imaging to determine the lowest value of TR that produced a sample temperature of 19°C with a change of no more than 1°C. To verify this, we chose to investigate the heating effects by applying the diffusion weighting gradients along the read, phase and slice directions individually rather than simultaneously. The diffusion weighting was identical to the subsequent imaging experiment (G = 0.5 T/m, δ = 3.5 ms, Δ = 8 ms, b‐value = 1498 s/mm^2^).

### Contrast Agent Concentration Optimization

To minimize steady state effects and ensure that the magnetization was near full relaxation, a tissue T_1_ value of approximately one fifth of the TR value was desired. To determine the contrast agent concentration that would provide this value, the T_1_ values from a previously acquired dataset 23 of CD‐1 embryos stained with 4–16 mM Gd‐DTPA were initially used as a benchmark. Building on this experience, *splotch* wild‐type embryos (*Pax3+/+*) were immersion‐fixed in 4% PFA doped with 2 mM Gd‐DTPA (Magnevist, Bayer‐Schering, Newbury, UK) (n = 1 per concentration), and stored at 4°C for 2 weeks. The T_1_ values of these embryos were measured approximately during preliminary scans using inversion recovery 3D spin‐echo acquisitions.

### Signal‐to‐Noise Optimization

#### Fixative Washout

Fixative washout was investigated as a possible method for increasing the SNR, as this has been shown to increase the tissue T_2_ with a much smaller increase on the tissue T_1_ 48. The embryo stained with 2 mM Gd‐DTPA was immersed in phosphate buffered saline (PBS) doped with 2 mM Gd‐DTPA at 4°C. T_1_ and T_2_ mapping data (matrix = 150 × 48 × 32, FOV = 15 × 9 × 6.4 to 15 × 9 × 7.2 mm, number of signals averaged [NSA] = 1) was acquired before PBS immersion and at 2, 5, and 7 weeks after PBS immersion using a 3D spin‐echo sequence (SE) (T_1_: TR = 500 ms, TE = 6.5 ms, TI = 50, 100, 150, 200, 250, 300, 350, 400 ms; and T_2_: TR = 500 ms, TE = 7.5, 12.5, 17.5, 22.5, 27.5, 32.5, 37.5, 42.5 ms). The mean T_1_ and T_2_ values were calculated for voxels within the spinal cord and cortex at each time point using in‐house software written in MATLAB (The Mathworks Inc., Natwick, MA). Also, 3D DW datasets (100 μm isotropic) were acquired before PBS immersion and 2 weeks after PBS immersion using a 3D fast spin‐echo (FSE) sequence with scanning parameters: matrix = 150 × 64 × 90, field of view (FOV) = 15 × 64 × 90 mm, NSA = 1, TR = 500 ms, effective TE = 14.75 ms, echo spacing (ESP) = 5.75 ms, echo train length (ETL) = 8, b‐value = 1498 s/mm^2^, G = 0.5 T/m, δ = 3.5 ms, Δ = 8 ms, 42 directions and 6 unweighted images, time = 4 h 48 min. The SNR was measured in the midbrain of each dataset for comparison.

#### MRI Sequence (3D FSE *versus* 3D SE)

Two widely available DW sequences, 3D FSE and 3D SE, were compared to assess which sequence provides the most appropriate combination of SNR, scan time and image quality. The same embryo (stained with 2 mM Gd‐DTPA) was used at five and seven weeks after immersion in PBS for the 3D SE and 3D FSE scans, respectively. The resolution, TR and diffusion parameters were set to be the same (75 μm isotropic, matrix = 200 × 96 × 120, FOV = 15 × 7.2 × 9 mm, TR = 500 ms, NSA = 1, G = 0.5 T/m, δ = 3.5 ms, Δ = 8 ms, b‐value = 1498 s/mm^2^, 42 directions and 6 unweighted images). TE was minimized for both acquisitions (3D SE: TE = 15.3 ms; and 3D FSE: effective TE = 14.75 ms, ESP = 5.75 ms, ETL = 4). The total acquisition times were 76 h 48 min and 19 h 12 min for the 3D SE and 3D FSE scans, respectively. The SNR was measured in the midbrain of datasets. To facilitate comparison of the SNR per unit time, the duration of the 3D FSE DW scan was assigned as one unit of time. Thus, the duration of the 3D SE DW scan was 4 units of time.

### Phenotypic Assessment

Using our optimized experimental procedure, we acquired DW images of a *splotch* homozygous embryo (*Pax3^Sp2H/Sp2H^*; on C57BL/6 background) and a wild‐type littermate (both immersion‐fixed in 4% paraformaldehyde doped with 2 mM Gd‐DTPA, followed by 2‐week immersion in PBS doped with Gd‐DTPA). The 3D DW images were corrected for movement with a rigid registration with three degrees of freedom (translation only) using the FLIRT registration tool 49, 50, which is part of the FMRIB Software Library (FSL v 4.1, Oxford, UK). Direction encoded color (DEC) maps were computed and visualized for comparison using MedINRIA (v1.9.4, Asclepios Project, France). For comparison with histology, the embryos were wax‐embedded, sectioned into 10‐μm sagittal slices and stained with hematoxylin and eosin (H&E). Additionally, reference images from the Schambra mouse embryo brain atlas 51 were used.

## RESULTS

### Tissue Temperature Equilibrium

**Figure 2:**
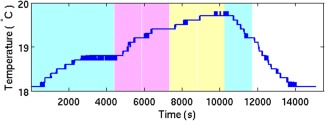
Temperature variation during test scans with TR = 500 ms. The background color denotes gradient direction: read, phase and slice are turquoise, pink, and orange, respectively. Gradient directions were applied sequentially. The temperature probe was in thermal contact with the sample container. TR = 500 ms, G = 0.5T/m, δ = 3.5 ms, Δ = 8 ms, b‐value = 1498 s/mm^2^.

Our preliminary experiments indicated that the MRI diffusion weighting gradients provided the main source of sample heating (temperature rises were only apparent for diffusion‐weighted scans). We determined that a minimum TR value of 500 ms was needed to maintain a stable sample temperature, which was important as diffusivity changes with temperature 47. Figure 2 shows that the temperature variation during test scans with identical imaging and diffusion weighting (G = 0.5 T/m, δ = 3.5 ms, Δ = 8 ms, b‐value = 1498 s/mm^2^) (applied sequentially in the read, phase and slice directions) stayed within 19 ± 1°C. Using this TR value for our short and long DW protocols (6 and 42 directions, respectively), the sample temperature remained stable throughout all imaging acquisitions (19 ± 1°C).

### Contrast Agent Concentration Optimization

Using preliminary measurements of stained *splotch* wild‐type embryos and previously acquired T_1_ data of stained CD‐1 embryos 23, we determined that 2 mM was the optimal concentration of Gd‐DTPA as it provided tissue T_1_ values (109 ms in the cortex and 104 ms in the spinal cord) that were approximately one fifth of the TR value (500 ms), which was necessary for minimizing steady state effects and ensuring the magnetization was fully relaxed.

### Signal‐to‐Noise Optimization

#### Fixative Washout

Following 2‐week immersion in PBS doped with Gd‐DTPA, we observed an approximate three‐fold increase in SNR. This was attained for each of the unweighted measurements (average SNR from 63 to 163) and each of the 42 directions (average SNR from 40 to 98). Additionally, PBS immersion resulted in an approximate two‐fold increase in the T_2_ values of the spinal cord and cortex, while the T_1_ values remained relatively constant (within 95% confidence intervals) (Table 1).

**Table 1:** T_1_ and T_2_ Values (and 95 % Confidence Intervals) in the Cortex and Spinal Cord Pre‐hydration, and 2, 5, and 7 Weeks post‐Fixative Washout

	Cortex	Spinal cord		
Study (n = 1)	T_1_ (ms) (95% CI)	T_2_ (ms) (95% CI)	T_1_ (ms) (95% CI)	T_2_ (ms) (95% CI)
Pre‐fixative washout	109 (100 and 120)	13 (13 and 13)	104 (96 and 114)	13 (13 and 13)
2 Weeks post‐fixative washout	105 (98 and 112)	27 (26 and 27)	101 (96 and 106)	25 (23 and 25)
5 Weeks post‐ fixative washout	110 (102 and 120)	31 (30 and 31)	107 (100 and 116)	27 (28 and 29)
7 Weeks post‐ fixative washout	111 (105 and 118)	30 (29 and 30)	106 (101 and 111)	29 (27 and 30)

#### MRI Sequence (3D FSE *versus* 3D SE)

**Figure 3:**
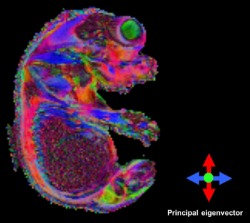
Representative slice from the DEC map generated from 3D SE data.

We observed a higher SNR per unit time (approximately three‐fold) using the 3D FSE sequence compared with the 3D SE sequence for both the unweighted measurements (average 58 versus 16) and 42 directions (average 31 versus 10). A comparison of line profiles between 3D SE and 3D FSE data indicated that the level of blurring in 3D FSE scans was acceptable (Supporting Fig. S1, which is available online). On visual inspection, there was very limited blurring in the DEC map generated from 3D FSE data compared with 3D SE data (Figs. 8c and 3, respectively).

### Phenotypic Assessment

**Figure 4:**
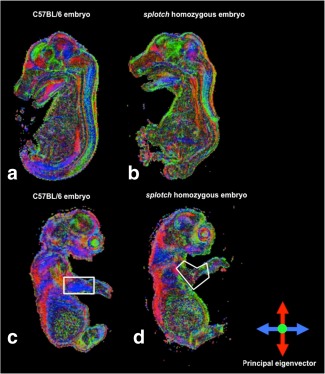
Mid‐sagittal view through DEC maps of a wild‐type (C57BL/6) embryo (**a,c**) and a splotch homozygous embryo (**b,d**) acquired using short DTI phenotyping protocol (six directions).

Using our short DW protocol (6 directions), we detected defects in the spinal cord of the *splotch* mutant embryo (Fig. 4) and a reduction in the FA of its limb muscles (Table 2) when compared with the wild‐type embryo. Additional differences in other areas of the central nervous system were indicative, prompting further investigation using a long DW protocol (42 directions).

**Table 2:** Fractional Anisotropy Values (Mean ± Standard Deviation) in a ROI in the Forelimb of Wild‐Type (C57BL/6) and Splotch Embryos for 6 and 30 Direction Diffusion μMRI Protocols

	Fractional anisotropy (arbitrary units)	
	6‐Direction protocol	42‐Direction protocol
Wild‐type (C57BL/6)	0.420 ± 0.132	0.410 ± 0.122
Splotch	0.284 ± 0.145	0.217 ± 0.123

**Figure 5:**
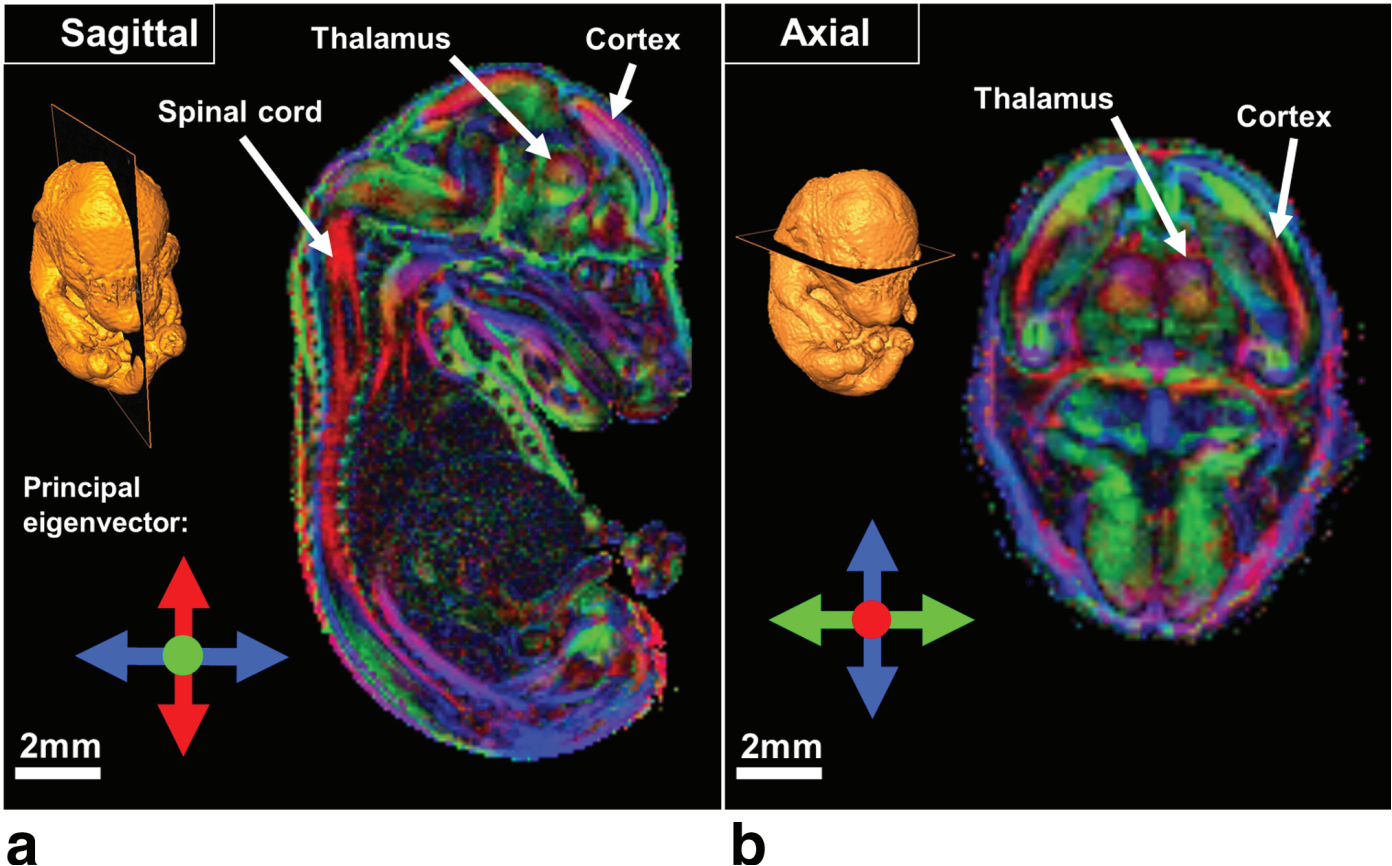
Sagittal (**a**) and axial (**b**) view through DEC maps of a wild‐type (C57BL/6) embryo acquired using extensive DTI phenotyping protocol (42 directions).

**Figure 6:**
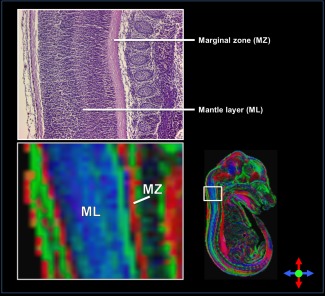
**a:** DEC map of a wild‐type (C57BL/6) embryo showing a sagittal view of the spinal cord. The eigenvectors indicate that the fibers of the marginal zone (MZ) and mantle layer (ML) of the spinal cord are in the ventral–dorsal and rostral–caudal directions, respectively, which correspond with the fiber orientation observed in the histology (**b**).

**Figure 7:**
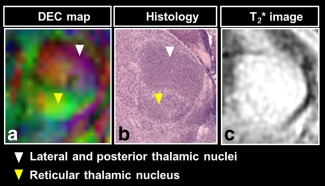
The DEC map of the thalamus (**a**) reveals the lateral and posterior thalamic nuclei (white arrow) and reticular thalamic nucleus (yellow arrow), which may be visualized using histology (**b**) 51, but is not apparent in a T_2_*‐weighted MR image (**c**) 23.

Our long DW protocol (42 directions) enabled clear visualization of several anatomical brain regions according to diffusion directionality in the wild‐type embryo, including the spinal cord, cortex, and thalamus (Fig. 5). The DEC maps of the wild‐type embryo indicated that the fibers of the marginal zone (MZ) and mantle layer (ML) of the spinal cord were oriented in the ventral–dorsal and rostral–caudal directions, respectively, which correlated with the histology (Fig. 6). Furthermore, the DEC maps elucidated rich microstructural information that is not evident using conventional T_2_*‐weighted MR imaging. For example, the thalamus appears homogenous in a representative T_2_*‐weighted MR image 23 (Fig. 7c), whereas the DEC maps revealed contrast within this structure (Fig. 7a) that corresponded to the lateral and posterior thalamic nuclei and the reticular thalamic nucleus (white and yellow arrows, respectively) in a histological slice [Fig. 7b, modified from the Schambra atlas 51].

**Figure 8:**
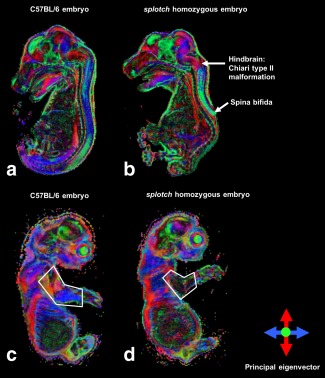
Mid‐sagittal view through DEC maps of a wild‐type (C57BL/6) embryo (**a,c**) and a splotch homozygous embryo (**b,d**) acquired using extensive DTI phenotyping protocol (42 directions). Phenotypic characteristics of the splotch mutant are highlighted (b,d).

Application of our methodology to the *splotch* mutant enabled phenotypic assessment of the whole embryo (Fig. 8). The primary spinal cord defect (spina bifida) could be clearly visualized in the DEC maps, and differences were evident in the overall brain size. Regions demarcated by the principal eigenvector, such as the pons, hindbrain, and midbrain, were readily identifiable, while the regional delineation in the thalamus was not as apparent in the mutant as the wild‐type. Strikingly, a clear herniation of the brain stem into the vertebral canal was visible, indicating the presence of the Chiari II malformation, which is a common finding in humans with spina bifida, but rarely described in previous studies of genetic mouse NTD models. Additionally, abnormal limb muscles were evident in the mutant compared with the wild‐type, as demonstrated by the reduction in FA (Table 2).

## DISCUSSION

In this study, we present two protocols for mouse embryo diffusion μMRI, together with guidelines for embryo preparation and imaging setup, which can be readily implemented to achieve a reasonable timeframe for data acquisition, making it suitable as a supplementary methodology for mouse embryo imaging and hopefully enabling more widespread use within the phenotyping community. We have demonstrated that DTI can provide sensitivity to tissue microstructure in the developing wild‐type mouse brain that is not apparent using conventional T_2_* relaxation‐based MRI approaches, in agreement with previous studies 24, 25, 26, 27, 30, 32, 34, 35. Subsequently, we successfully applied our protocols for investigation of the splotch mouse model of human NTDs, which enabled the first DTI assessment of spinal cord pathology in this model. Our findings suggest that microstructural differences between phenotypes may be elucidated using appropriate diffusion MRI protocols, particularly in the CNS and skeletal muscles.

To date, application of DTI for mouse embryo phenotyping has been limited due to the extensive data acquisition time involved in this methodology. While some embryo phenotyping studies have been carried out using DTI 33, 34, 35, widespread use will require guidelines for an approach that enables higher‐throughput screening and adaptability for different studies and research facilities. Our development brings us closer to this aim, allowing the user to balance the scan time, resolution, SNR, and b‐value according to their needs. For example, in our example study we chose a relatively low b‐value (1498 s/mm^2^) to reduce the total imaging time and maintain high resolution and SNR. Similarly, another study has recently been designed and optimized for ex vivo DW imaging of pig brains 52.

A wide array of diffusion μMRI protocols have been used in previous embryo phenotyping and developmental studies 24, 31, 32, 33, 34, 35, and there is currently no established methodology that ensures optimal data acquisition for any user. DTI data have typically been acquired using a 3D DW multiple SE sequence 53 in ∼ 15–30 h with 7–14 directions and 2–4 signal averages at ∼ 62 × 83 × 83 μm to 133 × 131 × 100 μm (12.5 dpc to adult mice). A 3D SE sequence has also been used providing DTI data in ∼ 32 h with seven directions and four signal averages at ∼ 78 × 94 × 94 μm (15.5 dpc) 26. More recently, Aggarwal et al implemented a 3D DW‐gradient and spin echo (GRASE) sequence 25 that enabled acquisition of DTI data in 1 h 50 min per direction at 60 μm isotropic (12 dpc).

Our example implementation produced an experimental procedure that enabled acquisition of DTI data in 24 min per direction at 75 μm isotropic using a standard 3D FSE sequence, such that preliminary or detailed screening of mid/late‐gestation embryos was possible in ∼3 h (with 6 directions plus one unweighted measurement) and ∼19 h (with 42 directions plus six unweighted measurements) per embryo, respectively, with no detectable instabilities introduced. While 3D SE sequences have been used more commonly in previous DTI studies, we found that a 3D FSE sequence provided higher SNR per unit time. A disadvantage of using FSE compared with SE is that blurring may be introduced. However, our protocols led to acceptable levels of blurring as the signal amplitudes from the first and last echo in the FSE echo train were comparable and we were able to shim the samples well. For this study, we also compared 3D‐SE and 3D‐FSE imaging modules as these are commonly available on preclinical systems. We used 3D FSE in our μDTI experimental procedure because of the comparable image quality and higher SNR per unit time compared with 3D SE. However, higher SNR per unit time sequences, such as GRASE 25 and SSFP 54, 55, could potentially be used with prior understanding of their possible artifacts.

Before data acquisition, we recommend optimization of several parameters, including the TR value (for tissue temperature equilibrium), contrast agent concentration (for optimal tissue T_1_ values) and the time required for fixative washout (for improved SNR). Earlier embryo phenotyping and developmental studies using DTI 24, 26, 32, 34, 35 immersion‐fixed embryos in a tissue fixative without a contrast agent, some of which 24, 32, 35 used fixative washout by means of PBS immersion for at least 24 h before imaging. More recent embryo studies have incorporated 0.1 mM 31 and 1 mM 25 Gd‐DTPA in the tissue fixation process (for more than 48 and 24 h, respectively), with no fixative washout. In our example, we determined that 2 mM of Gd‐DTPA followed by PBS immersion for 2 weeks was optimal for the experimental procedure required in our specific study.

The DEC maps acquired using our protocols provided microstructural information that was not apparent using conventional relaxation‐based MRI (Fig. 6), demonstrating the value of μDTI as a supplementary methodology for mouse embryo imaging. Visualization of the spinal cord tissue structure and thalamus using the DEC maps corresponded well with histology (Figs. 5 and 6), indicating that our methodology provided anatomically relevant information.

We were also able to detect additional differences within the mutant brain (Fig. 8), opening up new avenues for further investigation. For example, while studies of mouse genetic models of NTDs have been able to replicate many of the clinically observed defects, including spina bifida, craniorachischisis, and exencephaly 44, there have been very few reports in these mice of the Chiari type II malformation, in which the brainstem is elongated and the cerebellar vermis herniates through the foramen magnum. This disorder is commonly associated with spina bifida in humans 56, and yet the pathogenic basis of this link remains poorly understood. Our finding of the Chiari II malformation co‐existing with the open spina bifida in the genetic *splotch* mouse model offers a new opportunity to investigate the developmental basis of this malformation association. In the regions outside the CNS and skeletal muscles, we observed subtle DEC color differences, which is possibly due to gross differences in the orientation and position of regional anatomy of the organ systems.

With the creation of thousands of mutant mouse embryos facing researchers in the coming years, the challenge of phenotyping will require a sophisticated approach that uses existing and emerging techniques available in a complimentary way to maximize the information obtained during this “golden” period 57. Amongst these techniques, diffusion μMRI has the potential to offer unique information about tissue microstructure and connectivity in three dimensions. For example, a clinical study has shown that frontal white matter diffusion abnormalities may be detected using DTI, enabling diagnosis of mild traumatic brain injury in patients, which is not possible using MRI or CT 58. Thus, diffusion μMRI has the potential to reveal novel phenotypic characteristics related to microstructural changes that are not available using other techniques. However, it will be essential to compare the DTI data with existing and emerging techniques 57 to assess sensitivity and specificity to pathology and gauge its contribution to the field.

As demonstrated by our initial example, the protocols would enable users to develop an experimental procedure specific to their study and MRI system hardware. For example, shorter length scales could be probed using optimized gradient waveform SE sequences 59, 60, or more efficient gradient and sample cooling systems, which would allow a reduction in TR or higher b‐value. Also, studies at earlier developmental stages could allow for simultaneous imaging of multiple embryos owing to their smaller size, thus increasing the throughput. Further improvements could be made through the use of a dedicated mouse embryo imaging volume coil, which would provide better SNR.

## CONCLUSIONS

We have developed two protocols for whole‐body diffusion μMRI, which provided DTI data in 24 min per direction at 75 μm isotropic resolution, enabling preliminary and detailed screening in ∼ 3 h (6 directions plus one unweighted measurement) and ∼ 19 h (42 directions plus six unweighted measurements) per embryo, respectively. We determined that for our study, fixation with Gd‐DTPA followed by tissue rehydration enabled a combination of T_1_ reduction without excessive T_2_ shortening, providing increased SNR. Application of our protocols to the *splotch* mouse model of human NTDs provided assessment of the microstructural differences of the whole CNS between the wild‐type and mutant mouse. As our protocols can be readily applied to embryo imaging studies using a range of MRI hardware configurations, it may enable implementation of diffusion μMRI as a supplementary technique in mouse embryo screening pipelines and facilitate greater use within the phenotyping community. With the development of methods for automated analysis of DTI‐derived data 32, diffusion μMRI could prove a powerful tool for routine embryo phenotyping. Thus, we hope our guidelines will enable a wider adoption of diffusion μMRI for embryo phenotyping studies, which may provide new insights to congenital disease models.

## Supporting information

SUPPORTING FIG. S1. Line profile of a lateral–ventral line passing through the ocular globes for spin‐echo and fast spin‐echo scans with no diffusion weighting.

## References

[1] Collins FS , Rossant J , Wurst W . A mouse for all reasons. Cell 2007;128:9–13. 17218247 10.1016/j.cell.2006.12.018

[2] Nolan PM , Peters J , Strivens M , et al. A systematic, genome‐wide, phenotype‐driven mutagenesis programme for gene function studies in the mouse. Nat Genet 2000;25:440–443. 10932191 10.1038/78140

[3] Brown SD , Wurst W , Kuhn R , Hancock JM . The functional annotation of mammalian genomes: the challenge of phenotyping. Annu Rev Genet 2009;43:305–333. 19689210 10.1146/annurev-genet-102108-134143

[4] Copp AJ . Death before birth: clues from gene knockouts and mutations. Trends Genet 1995;11:87–93. 7732578 10.1016/S0168-9525(00)89008-3

[5] Adams D , Baldock R , Bhattacharya S , et al. Bloomsbury report on mouse embryo phenotyping: recommendations from the IMPC workshop on embryonic lethal screening. Dis Model Mech 2013;6:571–579. 23519032 10.1242/dmm.011833 PMC3634642

[6] Sharpe J , Ahlgren U , Perry P , Hill B , Ross A , Hecksher‐Sorensen J , Baldock R , Davidson D . Optical projection tomography as a tool for 3D microscopy and gene expression studies. Science 2002;296:541–545. 11964482 10.1126/science.1068206

[7] Walls JR , Coultas L , Rossant J , Henkelman RM . Three‐dimensional analysis of vascular development in the mouse embryo. PloS one 2008;3:e2853. 18682734 10.1371/journal.pone.0002853 PMC2478714

[8] Johnson JT , Hansen MS , Wu I , Healy LJ , Johnson CR , Jones GM , Capecchi MR , Keller C . Virtual histology of transgenic mouse embryos for high‐throughput phenotyping. PLoS Genet 2006;2:e61. 16683035 10.1371/journal.pgen.0020061 PMC1449902

[9] Degenhardt K , Wright AC , Horng D , Padmanabhan A , Epstein JA . Rapid 3D phenotyping of cardiovascular development in mouse embryos by micro‐CT with iodine staining. Circ Cardiovasc Imaging 2010;3:314–322. 20190279 10.1161/CIRCIMAGING.109.918482 PMC3059892

[10] Smith BR , Johnson GA , Groman EV , Linney E . Magnetic resonance microscopy of mouse embryos. Proc Natl Acad Sci USA 1994;91:3530–3533. 8170941 10.1073/pnas.91.9.3530 PMC43613

[11] Smith BR , Linney E , Huff DS , Johnson GA . Magnetic resonance microscopy of embryos. Comput Med Imaging Graph 1996;20:483–490. 9007215 10.1016/s0895-6111(96)00046-8

[12] Weninger WJ , Geyer SH , Mohun TJ , Rasskin‐Gutman D , Matsui T , Ribeiro I , Costa Lda F , Izpisua‐Belmonte JC , Muller GB . High‐resolution episcopic microscopy: a rapid technique for high detailed 3D analysis of gene activity in the context of tissue architecture and morphology. Anat Embryol 2006;211:213–221. 16429276 10.1007/s00429-005-0073-x

[13] Weninger WJ , Mohun T . Phenotyping transgenic embryos: a rapid 3‐D screening method based on episcopic fluorescence image capturing. Nat Genet 2002;30:59–65. 11743576 10.1038/ng785

[14] Bone SN , Johnson GA , Thompson MB . Three‐dimensional magnetic resonance microscopy of the developing chick embryo. Invest Radiol 1986;21:782–787. 3771148 10.1097/00004424-198610000-00003

[15] Pieles G , Geyer SH , Szumska D , et al. microMRI‐HREM pipeline for high‐throughput, high‐resolution phenotyping of murine embryos. J Anat 2007;211:132–137. 17532797 10.1111/j.1469-7580.2007.00746.x PMC2375802

[16] Schneider JE , Bose J , Bamforth SD , Gruber AD , Broadbent C , Clarke K , Neubauer S , Lengeling A , Bhattacharya S . Identification of cardiac malformations in mice lacking Ptdsr using a novel high‐throughput magnetic resonance imaging technique. BMC Dev Biol 2004;4:16. 15615595 10.1186/1471-213X-4-16 PMC545075

[17] Zhang X , Schneider JE , Portnoy S , Bhattacharya S , Henkelman RM . Comparative SNR for high‐throughput mouse embryo MR microscopy. Magn Reson Med 2010;63:1703–1707. 20512875 10.1002/mrm.22352

[18] Aguayo JB , Blackband SJ , Schoeniger J , Mattingly MA , Hintermann M . Nuclear magnetic resonance imaging of a single cell. Nature 1986;322:190–191. 3724861 10.1038/322190a0

[19] Petiet AE , Kaufman MH , Goddeeris MM , Brandenburg J , Elmore SA , Johnson GA . High‐resolution magnetic resonance histology of the embryonic and neonatal mouse: a 4D atlas and morphologic database. Proc Natl Acad Sci U S A 2008;105:12331–12336. 18713865 10.1073/pnas.0805747105 PMC2527911

[20] Cleary JO , Modat M , Norris FC , et al. Magnetic resonance virtual histology for embryos: 3D atlases for automated high‐throughput phenotyping. Neuroimage 2011;54:769–778. 20656039 10.1016/j.neuroimage.2010.07.039

[21] Jacobs RE , Ahrens ET , Dickinson ME , Laidlaw D . Towards a microMRI atlas of mouse development. Comput Med Imaging Graph 1999;23:15–24. 10091864 10.1016/s0895-6111(98)00059-7

[22] Zamyadi M , Baghdadi L , Lerch JP , Bhattacharya S , Schneider JE , Henkelman RM , Sled JG . Mouse embryonic phenotyping by morphometric analysis of MR images. Physiol Genom 2010;42A:89–95. 10.1152/physiolgenomics.00091.2010 PMC2957795 20682847

[23] Cleary JO , Price AN , Thomas DL , Scambler PJ , Kyriakopoulou V , McCue K , Schneider JE , Ordidge RJ , Lythgoe MF . Cardiac phenotyping in ex vivo murine embryos using microMRI. NMR Biomed 2009;22:857–866. 19598179 10.1002/nbm.1400

[24] Zhang J , Richards LJ , Yarowsky P , Huang H , van Zijl PC , Mori S . Three‐dimensional anatomical characterization of the developing mouse brain by diffusion tensor microimaging. Neuroimage 2003;20:1639–1648. 14642474 10.1016/s1053-8119(03)00410-5

[25] Aggarwal M , Mori S , Shimogori T , Blackshaw S , Zhang J . Three‐dimensional diffusion tensor microimaging for anatomical characterization of the mouse brain. Magn Reson Med 2010;64:249–261. 20577980 10.1002/mrm.22426 PMC2915547

[26] Mori S , Itoh R , Zhang J , Kaufmann WE , van Zijl PC , Solaiyappan M , Yarowsky P . Diffusion tensor imaging of the developing mouse brain. Magn Reson Med 2001;46:18–23. 11443706 10.1002/mrm.1155

[27] Berrios‐Otero CA , Wadghiri YZ , Nieman BJ , Joyner AL , Turnbull DH . Three‐dimensional micro‐MRI analysis of cerebral artery development in mouse embryos. Magn Reson Med 2009;62:1431–1439. 19859945 10.1002/mrm.22113 PMC2859666

[28] Petiet A , Johnson GA . Active staining of mouse embryos for magnetic resonance microscopy. Methods Mol Biol 2010;611:141–149. 19960328 10.1007/978-1-60327-345-9_11 PMC2811431

[29] Norris FC , Betts‐Henderson J , Wells JA , Cleary JO , Siow BM , Walker‐Samuel S , McCue K , Salomoni P , Scambler PJ , Lythgoe MF . Enhanced tissue differentiation in the developing mouse brain using magnetic resonance micro‐histology. Magn Reson Med 2013;70:1380–1388. 23213043 10.1002/mrm.24573

[30] Basser PJ , Mattiello J , LeBihan D . MR diffusion tensor spectroscopy and imaging. Biophys J 1994;66:259–267. 8130344 10.1016/S0006-3495(94)80775-1 PMC1275686

[31] Chuang N , Mori S , Yamamoto A , et al. An MRI‐based atlas and database of the developing mouse brain. NeuroImage 2011;54:80–89. 20656042 10.1016/j.neuroimage.2010.07.043 PMC2962762

[32] Zhang J , Miller MI , Plachez C , Richards LJ , Yarowsky P , van Zijl P , Mori S . Mapping postnatal mouse brain development with diffusion tensor microimaging. Neuroimage 2005;26:1042–1051. 15961044 10.1016/j.neuroimage.2005.03.009

[33] Andrews W , Liapi A , Plachez C , Camurri L , Zhang J , Mori S , Murakami F , Parnavelas JG , Sundaresan V , Richards LJ . Robo1 regulates the development of major axon tracts and interneuron migration in the forebrain. Development 2006;133:2243–2252. 16690755 10.1242/dev.02379

[34] Wang Y , Zhang J , Mori S , Nathans J . Axonal growth and guidance defects in Frizzled3 knock‐out mice: a comparison of diffusion tensor magnetic resonance imaging, neurofilament staining, and genetically directed cell labeling. J Neurosci 2006;26:355–364. 16407530 10.1523/JNEUROSCI.3221-05.2006 PMC6674392

[35] Zhang J , Chen YB , Hardwick JM , Miller MI , Plachez C , Richards LJ , Yarowsky P , van Zijl P , Mori S . Magnetic resonance diffusion tensor microimaging reveals a role for Bcl‐x in brain development and homeostasis. J Neurosci 2005;25:1881–1888. 15728827 10.1523/JNEUROSCI.4129-04.2005 PMC6726064

[36] Jones DK . The effect of gradient sampling schemes on measures derived from diffusion tensor MRI: a Monte Carlo study. Magn Reson Med 2004;51:807–815. 15065255 10.1002/mrm.20033

[37] Tournier JD , Calamante F , Gadian DG , Connelly A . Direct estimation of the fiber orientation density function from diffusion‐weighted MRI data using spherical deconvolution. Neuroimage 2004;23:1176–1185. 15528117 10.1016/j.neuroimage.2004.07.037

[38] Alexander DC . Maximum entropy spherical deconvolution for diffusion MRI. In: Christensen GE, Sonka M, editors. Heidelberg: Springer; 2005. 10.1007/11505730_7 17354686

[39] Zhang H , Schneider T , Wheeler‐Kingshott CA , Alexander DC . NODDI: practical in vivo neurite orientation dispersion and density imaging of the human brain. Neuroimage 2012;61:1000–1016. 22484410 10.1016/j.neuroimage.2012.03.072

[40] Peled S , Yeshurun Y . Superresolution in MRI: application to human white matter fiber tract visualization by diffusion tensor imaging. Magn Reson Med 2001;45:29–35. 11146482 10.1002/1522-2594(200101)45:1<29::aid-mrm1005>3.0.co;2-z

[41] Scherrer B , Gholipour A , Warfield SK . Super‐resolution in diffusion‐weighted imaging. Med Image Comput Comput Assist Interv 2011;14(Pt 2):124–132. 21995021 10.1007/978-3-642-23629-7_16 PMC3687082

[42] Raffelt D , Tournier JD , Rose S , Ridgway GR , Henderson R , Crozier S , Salvado O , Connelly A . Apparent fibre density: a novel measure for the analysis of diffusion‐weighted magnetic resonance images. Neuroimage 2012;59:3976–3994. 22036682 10.1016/j.neuroimage.2011.10.045

[43] Copp AJ , Brook FA , Estibeiro JP , Shum AS , Cockroft DL . The embryonic development of mammalian neural tube defects. Prog Neurobiol 1990;35:363–403. 2263736 10.1016/0301-0082(90)90037-h

[44] Greene ND , Stanier P , Copp AJ . Genetics of human neural tube defects. Hum Mol Genet 2009;18(R2):R113–R129. 19808787 10.1093/hmg/ddp347 PMC2758708

[45] Greene ND , Massa V , Copp AJ . Understanding the causes and prevention of neural tube defects: insights from the splotch mouse model. Birth Defects Res A Clin Mol Teratol 2009;85:322–330. 19180568 10.1002/bdra.20539

[46] Epstein DJ , Vekemans M , Gros P . Splotch (Sp2H), a mutation affecting development of the mouse neural tube, shows a deletion within the paired homeodomain of Pax‐3. Cell 1991;67:767–774. 1682057 10.1016/0092-8674(91)90071-6

[47] Holz M , Heil SR , Sacco A . Temperature‐dependent self‐diffusion coefficients of water and six selected molecular liquids for calibration in accurate 1H NMR PFG measurements Phys Chem Chem Phys 2000;2:4740–4742.

[48] Shepherd TM , Thelwall PE , Stanisz GJ , Blackband SJ . Aldehyde fixative solutions alter the water relaxation and diffusion properties of nervous tissue. Magn Reson Med 2009;62:26–34. 19353660 10.1002/mrm.21977 PMC3188415

[49] Jenkinson M , Bannister P , Brady M , Smith S . Improved optimization for the robust and accurate linear registration and motion correction of brain images. Neuroimage 2002;17:825–841. 12377157 10.1016/s1053-8119(02)91132-8

[50] Jenkinson M , Smith S . A global optimisation method for robust affine registration of brain images. Medical Image Anal 2001;5:143–156. 10.1016/s1361-8415(01)00036-6 11516708

[51] Schambra UB , Lauder JM , Silver J . Atlas of the prenatal mouse brain. San Diego: Academic Press Inc; 1992. 10.1016/0014-4886(91)90034-a 1748192

[52] Dyrby TB , Baare WF , Alexander DC , Jelsing J , Garde E , Sogaard LV . An ex vivo imaging pipeline for producing high‐quality and high‐resolution diffusion‐weighted imaging datasets. Hum Brain Mapp 2011;32:544–563. 20945352 10.1002/hbm.21043 PMC6870191

[53] Mori S , van Zijl PC . A motion correction scheme by twin‐echo navigation for diffusion‐weighted magnetic resonance imaging with multiple RF echo acquisition. Magn Reson Med 1998;40:511–516. 9771567 10.1002/mrm.1910400403

[54] McNab JA , Miller KL . Sensitivity of diffusion weighted steady state free precession to anisotropic diffusion. Magn Reson Med 2008;60:405–413. 18666106 10.1002/mrm.21668

[55] Miller KL , McNab JA , Jbabdi S , Douaud G . Diffusion tractography of post‐mortem human brains: optimization and comparison of spin echo and steady‐state free precession techniques. Neuroimage 2012;59:2284–2297. 22008372 10.1016/j.neuroimage.2011.09.054 PMC3314951

[56] Harding BN , Copp AJ . Malformations In: Love S, Louis DN, Ellison DW, editors. Greenfield's neuropathology, 8th ed. London: Hodder Arnold; 2008 p 335–479.

[57] Norris FC , Wong MD , Green ND , Scambler PJ , Weaver T , Weninger WJ , Mohun TJ , Henkelman RM , Lythgoe MF . A coming of age: advanced imaging technologies for characterising the developing mouse. Trends Genet 2013;29:700–711. 24035368 10.1016/j.tig.2013.08.004

[58] Lipton ML , Gulko E , Zimmerman ME , Friedman BW , Kim M , Gellella E , Gold T , Shifteh K , Ardekani BA , Branch CA . Diffusion‐tensor imaging implocates prefrontal axonal injury in executive function impairment following very mild traumatic brain injury. Radiology 2009;252:816–824. 19567646 10.1148/radiol.2523081584

[59] Drobnjak I , Siow B , Alexander DC . Optimizing gradient waveforms for microstructure sensitivity in diffusion‐weighted MR. J Magn Reson 2010;206:41–51. 20580294 10.1016/j.jmr.2010.05.017

[60] Siow B , Drobnjak I , Chatterjee A , Lythgoe MF , Alexander DC . Estimation of pore size in a microstructure phantom using the optimised gradient waveform diffusion weighted NMR sequence. J Magn Reson 2012;214:51–60. 22116034 10.1016/j.jmr.2011.10.004

