# Supplementary figures and images for: Diffusion microscopic MRI of the mouse embryo: Protocol and practical implementation in the splotch mouse model
Source: Magn Reson Med. 2014 Mar 13;73(2):731–9. doi: 10.1002/mrm.25145 (PMC4737188; doi:10.1002/mrm.25145)

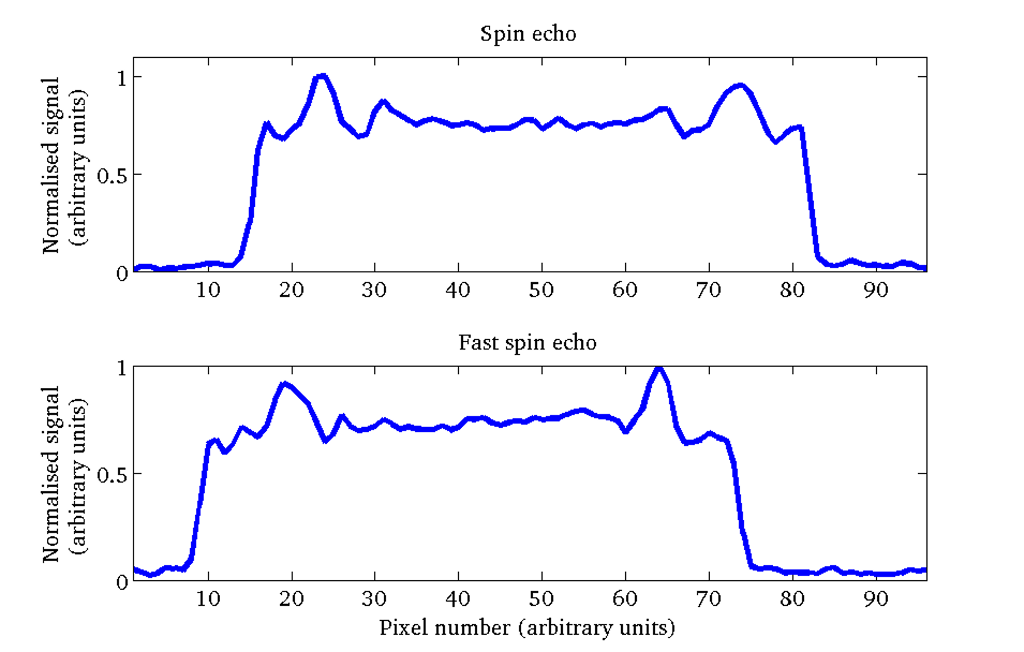

Supplement: Supplementary file 1 — SUPPORTING FIG. S1. Line profile of a lateral–ventral line passing through the ocular globes for spin‐echo and fast spin‐echo scans with no diffusion weighting. [file MRM-73-731-s001.tif]
